# Safety, tolerability, pharmacokinetics and pharmacokinetic-pharmacodynamic modeling of cetagliptin in patients with type 2 diabetes mellitus

**DOI:** 10.3389/fendo.2024.1359407

**Published:** 2024-03-11

**Authors:** Chen Zhou, Sufeng Zhou, Jie Wang, Lijun Xie, Zhanhui Lv, Yuqing Zhao, Lu Wang, Huan Luo, Daosheng Xie, Feng Shao

**Affiliations:** ^1^ Phase I Clinical Trial Unit, the First Affiliated Hospital with Nanjing Medical University, Nanjing, China; ^2^ Department of Clinical Pharmacology, Pharmacy College, Nanjing Medical University, Nanjing, China; ^3^ Department of Clinical Pharmacy, School of Basic Medicine and Clinical Pharmacy, China Pharmaceutical University, Nanjing, China; ^4^ Clinical Development Department, Beijing Sun-novo Pharmaceutical Research Co., Ltd, Beijing, China; ^5^ Clinical Development Department, Beijing Noahpharm Medical Technology Co., Ltd, Beijing, China

**Keywords:** cetagliptin, dipeptidyl peptidase-4, pharmacokinetics, pharmacodynamics, type 2 diabetes mellitus

## Abstract

**Aims:**

To evaluate the safety, tolerability, pharmacokinetics (PK), and pharmacodynamics (PD) of cetagliptin (CAS number:2243737-33-7) in Chinese patients with type 2 diabetes mellitus (T2DM). A population PK/PD model was developed to quantify the PK and PD characteristics of cetagliptin in patients.

**Materials and methods:**

32 Chinese adults with T2DM were enrolled in this study. The subjects were randomly assigned to receive either cetagliptin (50 mg or 100 mg), placebo, or sitagliptin (100 mg) once daily for 14 days. Blood samples were collected for PK and PD analysis. Effects on glucose, insulin, C-peptide, and glucagon were evaluated following an oral glucose tolerance test (OGTT) (day15). Effects on HbA1c and glycated albumin (GA), and safety assessments were also conducted. Meanwhile, a population PK/PD model was developed by a sequential two-step analysis approach using Phoenix.

**Results:**

Following multiple oral doses, cetagliptin was rapidly absorbed and the mean half-life were 34.9-41.9 h. Steady-state conditions were achieved after 1 week of daily dosing and the accumulation was modest. The intensity and duration of DPP-4 inhibition induced by 50 mg cetagliptin were comparable with those induced by sitagliptin, and 100 mg cetagliptin showed a much longer sustained DPP-4 inhibition (≥80%) than sitagliptin. Compared with placebo group, plasma active GLP-1 AUEC_0-24h_ increased by 2.20- and 3.36-fold in the 50 mg and 100 mg cetagliptin groups. A decrease of plasma glucose and increase of insulin and C-peptide were observed following OGTT in cetagliptin groups. Meanwhile, a tendency of reduced GA was observed, whereas no decreasing trend was observed in HbA1c. All adverse events related to cetagliptin and sitagliptin were assessed as mild. A population PK/PD model was successfully established. The two-compartment model and Sigmoid-E_max_ model could fit the observed data well. Total bilirubin (TBIL) was a covariate of volume of peripheral compartment distribution (V_2_), and V_2_ increased with the increase of TBIL.

**Conclusions:**

Cetagliptin was well tolerated, inhibited plasma DPP-4 activity, increased plasma active GLP-1 levels, and exhibited a certain trend of glucose-lowering effect in patients with T2DM. The established population PK/PD model adequately described the PK and PD characteristics of cetagliptin.

## 1 Introduction

The incidence of Diabetes mellitus (DM) continues to rise globally, posing a major threat to global health (1–3). Globally, about 1 in 11 adults has diabetes [90% have type 2 diabetes (T2DM)], and Asia is the center of the global T2DM epidemic. China and India are the first two epicenters (1). DM is a chronic metabolic disorder characterized by insufficient insulin production and/or insulin resistance caused by environmental and genetic factors (4). Hyperglycemia is a typical clinical manifestation of DM. Chronic hyperglycemia can lead to microvascular and macrovascular complications (5, 6). These chronic complications seriously impact the patient’s quality of life (2, 7–9). The data indicated that patients with DM had approximately three times higher of hospitalization rates for cardiovascular disease, twelve times higher for end-stage renal disease, and twenty times higher for non-traumatic lower extremity amputation compared to patients without DM (10).

Effective control of blood glucose levels is the main goal of DM treatment. However, it also brings the risk of treatment-related hypoglycemia. Hypoglycemia has always been considered a dangerous side effect of the treatment of DM with insulin or insulin secretagogues (11, 12). Studies have shown that hypoglycemia is associated with an increased risk of cardiovascular events and mortality (11). A relatively early epidemiological study reported that hypoglycemia caused 4% of the deaths of DM patients under the age of 50 (13). A recent Norwegian study found that hypoglycemia was directly responsible for a greater mortality risk. Patients with type 1 diabetes under the age of 56 have a mortality rate that was above 8% (14). These findings emphasized the importance of carefully balancing the benefits and potential harms for DM patients treated with insulin or insulin secretagogues (11).

Dipeptidyl peptidase 4 inhibitor (DPP-4i) is an oral hypoglycemic agent with specific benefits for the treatment of DM and a low risk of hypoglycemia (15, 16). It can highly and selectively inhibit DPP-4 enzyme activity. The inhibitors can prevent the breakdown of the incretins, glucagon-like peptide-1 (GLP-1) and glucose-dependent insulinotropic peptide (17). Among them, GLP-1 is believed to mediate the main therapeutic effect of DPP-4i (18, 19). GLP-1 induces insulin secretion to reduce blood glucose in a glucose-dependent manner, via activating GLP-1 receptors on the β-cell (12, 20–22). It can also inhibit α-cell secretion of glucagon to further reduce blood glucose (20, 23). Moreover, GLP-1 can reduce appetite, weaken gastrointestinal motility, delay gastric emptying, enhance satiety to effectively control weight, and help control blood glucose (22, 24). Generally, DPP-4is are well tolerated, have a low risk of hypoglycemia and weight gain, and are expected to have long-term beneficial effects on β-cell function and quality (18, 25). DPP-4 inhibitors on the market include sitagliptin (the first DPP-4i), vildagliptin, saxagliptin, linagliptin and alogliptin. Sitagliptin is an orally effective DPP-4 inhibitor, used as the positive control drug in this study. In healthy male subjects, sitagliptin exhibited approximately 80% or greater inhibition of DPP-4 activity and increased postprandial active GLP-1 levels without causing hypoglycemia (26). And in patients with type 2 diabetes, sitagliptin significantly reduced levels of glycated hemoglobin without causing weight gain and hypoglycemia (27).

Cetagliptin (CAS number:2243737-33-7) is a novel and highly selective DPP-4i intended for the treatment of T2DM (28–30). Preclinical studies (data not published) showed that cetagliptin could significantly inhibit blood glucose levels and serum DPP-4 activity in Zucker Diabetic Fatty rats (≧80%), and the inhibitory activity on DPP-4 was stronger than sitagliptin (25). The first-in-human phase I clinical studies also showed that cetagliptin could inhibit the active of DPP-4, increased the levels of active GLP-1, and had good tolerability with no dose-limiting toxicity observed after single oral doses of 12.5 to 400 mg of cetagliptin in healthy subjects (30). In addition, a study evaluating the pharmacokinetics (PK), pharmacodynamics (PD), safety, and tolerability of cetagliptin following multiple oral doses in healthy subjects demonstrated that a dose regimen of once-daily oral dose of ≧50 mg of cetagliptin resulted in sustained DPP-4 inhibition (≧80%), increased active GLP-1 levels, and decreased blood glucose levels. All the aforementioned preclinical and clinical results indicated cetagliptin has significant potential for the treatment of T2DM (31).

However, we lack the safety, PK, and PD profiles of cetagliptin in patients with T2DM. For this reason, we report here this study to initially evaluate the safety, PK, and PD characteristics of cetagliptin, compared with sitagliptin, after fasting oral administrations in patients with T2DM. Meanwhile, a population PK/PD model was established to describe the population PK and PD characteristics of cetagliptin in T2DM patients, and the effects of demographic characteristics and clinical variables on the PK and PD were evaluated.

## 2 Materials and methods

### 2.1 Study participants

A total of 32 Chinese adults with T2DM were enrolled in this study. Patients included in the study were newly diagnosed with T2DM based on the diagnostic criteria and classification established by the World Health Organization (WHO) in 1999 and had not received any hypoglycemic drugs; or patients were diagnosed with T2DM and were currently controlled by diet and exercise and had not taken any hypoglycemic drugs in the past 12 weeks (13); Patients aged 18 to 65 years old; and both males and females in each dose group; Male weight ≥50.0 kg, female weight ≥45.0 kg, body mass index (BMI) 19.00-30.00 kg/m^2^; 6.5%≤HbA1c< 9% and fasting blood glucose<13.4 mmol/L. Subjects were excluded if they had a history of pancreatic injury or pancreatitis, significant diabetic complications, type 1 diabetes, gestational diabetes, special type diabetes, past severe hypoglycemic events, liver and kidney dysfunction, poor blood pressure and lipid control, and allergic to DPP-4i.

### 2.2 Study design

This study was conducted at the First Affiliated Hospital of Nanjing Medical University (Nanjing, China) and approved by the Ethics Committees of the hospital. It was registered at: http://www.chinadrugtrials.org.cn/index.html (CTR20190599). Principles of Declaration of Helsinki, Good Clinical Practice, and International Conference for Harmonization were adhered to during the conduct of this study. All subjects signed written informed consent prior to being screened for eligibility.

This was a single-center, randomized, double-blind, placebo and positive-controlled, single and multiple oral-dose study. A total of 32 Chinese adults diagnosed with T2DM were recruited for this study and allocated into two dosage groups: 50 mg and 100 mg, each consisting of 16 participants. Within each dosage group, the sixteen subjects were randomly assigned in a ratio of 10:2:4 to receive either cetagliptin (at doses of either 50 mg or 100 mg), a placebo that matched the active drug, or a positive control (sitagliptin at a dose of 100 mg). The positive control was designed as open label.

Eligible subjects were admitted to the study site on day -3, and then completed the baseline examination and an oral glucose tolerance test (OGTT) on day -2 and day -1, respectively. Subjects underwent medication randomization on day -1, and were assigned the corresponding investigational products. They were orally administered to the drug once every morning on fasting condition for 14 consecutive days. On day 1 and day 14, drinking water was not allowed from 1 h before dosing until 2 h post dose. Subjects were remained fasted for 4 h post dose and standard meals were provided at 4 h and 10 h post-dose. While, on day 2 to day 13, drinking water was not allowed from 1 h before dosing until 1 h post dose, standard meals were provided at 1 h, 4 h, and 10 h post-dose. After finished the dosing, another OGTT was performed on day 15. Blood samples were collected at designated time points for the analysis of PK/PD and exploratory indicators. Subjects were discharged after completion of the safety assessments on day 19.

### 2.3 PK analysis

#### 2.3.1 Sample collection for PK analysis

Blood samples for PK analysis were collected within 0.5 hours before dosing, 0.5, 1, 2, 3, 4, 5, 6, 8, 12 and 24 h after dosing on day 1; within 0.5 hours before dosing on day 7 and day 10; within 0.5 h before dosing and 0.5, 1, 2, 3, 4, 5, 6, 8, 12, 24, 48, 72, 96 and 120 h after dosing (blood samples for sitagliptin group were not collected at 72 h, 96 h and 120 h) on day 14. At each blood sampling point, 3 mL of blood samples were collected into centrifuge tubes containing anticoagulant (K_2_EDTA) and centrifuged at 1500 g, 2-8°C for 10 min, the plasma samples were separated and stored at -70 ± 10°C until analysis. Plasma concentrations of cetagliptin and sitagliptin were determined using validated liquid chromatography-tandem mass spectrometry methods. For cetagliptin and sitagliptin, the linear calibration ranges were 0.5-5000 ng/mL and 1-800 ng/mL, respectively (31).

#### 2.3.2 PK analysis

PK parameters were calculated using non-compartmental analysis with Phoenix WinNonlin (version 8.1, Certara, Co., Princeton, NJ, United States). Peak plasma concentration after administration (C_max_) and time to reach C_max_ (T_max_) were obtained directly from the observed data, elimination half-life (t_1/2_) was calculated as ln2/λz using the best fit mode, where λz was the terminal elimination rate constant. Area under the plasma concentration-time curve from zero to the last measurable concentration (AUC_0-t_) was estimated using the linear trapezoidal method and AUC from zero to infinity (AUC_0-∞_) was calculated as AUC_0-t_+C_t_/λ_z_, where C_t_ was the last measured concentration. The average value of the steady-state concentration (C_av, ss_) was calculated as AUC_0-τ_/τ (τ=24h). Apparent total plasma clearance after non-intravenous (CL/F) and apparent volume of distribution in terminal phase after non-intravenous (V_z_/F) were calculated as Dose/AUC_0-∞_ and CL/λ_z_, respectively. Accumulation ratios of C_max_ (R_Cmax_) and AUC (R_AUC0-24h_) were calculated as C_max, day 14_/C_max, day 1_ and AUC_0-τ, day 14_/AUC_0-24 h, day 1_.

### 2.4 PD analysis

#### 2.4.1 DPP-4 inhibition and PK-PD relationship

Blood samples for DPP-4 activity determination were collected at the same time points as for PK. 1 mL blood samples were collected into blood-collecting tubes containing K_2_-EDTA. The tubes were placed on ice until centrifugation. The blood samples were centrifuged at 2-8°C, 1500 g for 10 min within 1 hour after blood collection. After centrifugation, the supernatant were evenly divided into two aliquots and stored at -80 ± 10°C. The plasma DPP-4 activity was determined using a fluorescent method with the substrate Gly-Pro-7-amide-4-methylcoumarin. A range of 3-400 µM was covered by the linear calibration. For the 80-120% range, the relative error of accuracy was met (31).

The degree of inhibition of DPP-4 enzyme activity relative to baseline (DPP-4 inhibition, %) after administration was calculated as the following equation:


$$\text{DPP}-4\text{ inhibition }\left(\%\right)=\left(1-\frac{\text{DPP}-4\text{ activity}}{\text{DPP}-4\text{ activity},\text{Baseline}}\right)\times 100$$

The calculated PD parameters for evaluation of DPP-4 inhibition were as follows: maximum observed response (R_max_), the time of maximum observed response (T_Rmax_), area under the effect curve from the time of dosing to 24 h or the last measurable response (AUEC_0-24 h_, AUEC_0-t_), the duration for DPP-4 inhibition rate of >80% (DUR_80%_), the observed effect at 24 h postdose (E_24 h_) and minimum observed response (R_min_) on day 14.

Furthermore, a maximum inhibitory efficacy (E_max_) model was used to evaluate the relationship between plasma concentrations (cetagliptin or sitagliptin) and DPP-4 inhibition. E_max_ and the plasma concentration of cetagliptin or sitagliptin that produced half the maximum effect (EC_50_) were provided using Phoenix WinNonlin software version 8.1.

#### 2.4.2 GLP-1 activity

Blood samples for GLP-1 activity evaluation were collected at the same time points as for PK. 2 mL blood samples were collected for GLP-1 activity detection (20 μL DPP-4i was added to blood-collecting tubes beforehand). The tubes were placed on ice until centrifugation. The blood samples were centrifuged at 2-8°C, 1500 g for 10 minutes within 1 hour after blood collection. After centrifugation, the blood samples were evenly divided into two aliquots and stored at -80 ± 10°C. Plasma active GLP-1 concentrations were determined using a validated ELISA method. Linear calibration curves were obtained in the concentration range of 0.017-276 pM (31).

The change of GLP-1 concentration from baseline (△GLP-1) was calculated as follows:


Δ [GLP-1] = [GLP-1] (t) - [GLP-1] (0)

The calculated parameters for GLP-1 activity were as follows: the baseline GLP-1 value before dosing (Baseline), R_max_, T_Rmax_, AUEC_0-t_, AUEC_0-24h_, GLP-1 concentration change at 2 h after lunch (△GLP-1-6h), GLP-1 concentration change at 2 h after dinner (△GLP-1-12h), R_min_ on day 14, and the average response on day 14 (R_avg_).

#### 2.4.3 Effects on glucose, insulin, C-peptide, and glucagon

To assess the impact of investigational products on glucose, insulin, C-peptide, and glucagon, OGTT tests were conducted on day -1 and day 15 following a fasting period of more than 8 hours. Subjects received a 75 g oral glucose dose and blood samples (1.5 mL for glucose, 3.5 mL for insulin and C-peptide, 2 mL for glucagon) were collected at 0, 0.167, 0.5, 1, 1.5, 2, and 3 h after ingestion of glucose. The PD parameters (AUEC_0-t_) for glucose, insulin, C-peptide, and glucagon were calculated by using drug effect module of non-compartmental method with Phoenix WinNonlin.

### 2.5 Preliminary efficacy evaluation

Blood samples for determination of fasting plasma glucose (FPG) and 2 hour postprandial plasma glucose (2 h PPG) were collected on day -2, day 7 and day 14. Additionally, blood samples for determination of glycated Hemoglobin A1C (HbA1c) and glycated Albumin (GA) were collected on day -2 and day 14. The changes of the above indexes relative to pre-treatment baseline (day -2) were evaluated and compared to investigate the preliminary efficacy of investigational product.

### 2.6 Safety and tolerability assessments

Safety and tolerability were evaluated by monitoring adverse events (AEs), laboratory tests (including blood routine, urine routine, stool routine, blood biochemical test and coagulation function), vital signs, physical examination, 12-lead electrocardiogram and other indicators. AEs were monitored and collected throughout the study. Descriptive analysis of the type and intensity of AEs were conducted according to NCICTC AE5.0.

### 2.7 Development and evaluation of population PK/PD model

A population PK/PD (PopPK/PD) model was developed to describe the relationship between cetagliptin and DPP-4 inhibition. A sequential two-step analysis approach to modeling building was implemented. First, a population PK model was developed, and then parameters were fixed to establish the PopPK/PD model. The nonlinear mixed effect modeling method was used to establish the PopPK/PD model. Model selection criteria were based on goodness-of-fit plots, objective function value (OFV, equal to −2 log-likelihood), Akaike information criteria (AIC), and precision of parameter estimates.

#### 2.7.1 Development of PopPK model

A total of 560 plasma concentrations of cetagliptin from 32 patients with T2DM were used for PopPK analysis. The structural model was tested using either one- or two-compartment PK models. Individual variation was modeled using an exponential form (Equation 1):


(1) $${\text{P}}_{\text{ij}}={\text{P}}_{\text{j}}\times {\text{e}}^{{\text{η}}_{\text{ij}}}$$

P_j_ represents the typical value of the jth parameter in population and Pij represents the true value of a parameter for the ith subject on the jth parameter. The inter-individual variability ( η ) of PK parameters was assumed to follow a log-normal distribution with a mean of 0 and a variance in ${\text{ω}}^{2}$ .

The additive error model (Equation 2), proportional error model (Equation 3), and additive and proportional error model (Equation 4) were evaluated to describe the residual variability:


(2) $${\text{C}}_{\text{ij}}={\text{IPERD}}_{\text{ij}}+{\text{ε}}_{\text{ij}}$$

(3) $${\text{C}}_{\text{ij}}={\text{IPERD}}_{\text{ij}}\times \left(1+{\text{ε}}_{\text{ij}}\right)$$

(4) $${\text{C}}_{\text{ij}}={\text{IPERD}}_{\text{ij}}\times \left(1+{\text{ε}}_{\text{ij},1}\right)+{\text{ε}}_{\text{ij},2}$$

Where C_ij_ is the observation concentration of the ith subject at the jth sampling point and IPRED_ij_ is the subject’s prediction value. The residual variability ( ε ) is normally distributed with a mean of 0 and a variance in $\sigma^{2}$ .

The stepwise forward inclusion/backward elimination approach was used to investigate the covariate effects on PopPK parameters.

#### 2.7.2 Development of PopPKPD model

As a result of data with an absolute value of CWRES greater than 5 being excluded, only 554 blood concentrations of cetagliptin were included in development of the PopPK/PD model. A direct-effect model was used to build the PK/PD model; and the model formula was as follows:


$$\text{DPP}-4\text{ inhibition }\left(\%\right)=E_{max}*C^{\gamma }/\left(EC_{50}^{\gamma }+C^{\gamma }\right)$$

Where $E_{max}$ = maximum DPP-4 inhibition (%); C = plasma concentration; $EC_{50}$ = plasma concentration of cetagliptin that achieves 50% of the maximum drug effect; and γ = hill coefficient, which describes the steepness of the concentration-response curve.

Interindividual variation and residual variability were considered the same as the PopPK model.

#### 2.7.3 Model evaluation

Goodness-of-fit plots were assessed to describe the adequacy of the final PopPK/PD model, including observations vs. population predictions, observations vs. individual predictions, conditional weighted residuals (CWRES) vs. population predictions, and CWRES vs. time., A bootstrap resampling procedure was performed to assess the stability of the final PopPK/PD model. A total of 1000 bootstrap datasets were generated by random sampling with replacement, and the PK parameters were re-estimated using the final population model. The median parameter value and their 95% confidence intervals (95% CIs) from bootstrap estimates were compared using the estimates of the final model. In addition, a visual predictive check (VPC) was used to assess the predictive ability of the final model. A total of 1000 simulations of the final population PK model were performed. The VPC graphically showed the observations and different percentiles of simulated concentrations (5th, median, and 95th percentiles).

### 2.8 Statistical analysis

Descriptive statistics of subject demographics were summarized using mean and standard deviation (SD) or number and percentage. The safety assessments after administration were summarized descriptively or listed. All the PK and PD parameters were expressed as mean and SD or median and range (T_max_). Analysis of the PD parameters was performed using ANOVA.

## 3 Results

### 3.1 Subject demographics

A total of 32 Chinese subjects with T2DM, comprising 22 males and 10 females, were enrolled and randomly assigned to three groups in this study: the cetagliptin group (n=20), the placebo group (n=4), and the sitagliptin group (n=8). All subjects completed the study as planned and included in the safety and PD analysis, and 28 subjects of them who received cetagliptin or sitagliptin were included in the PK analysis. There were no statistically significant differences in demographic characteristics between treatment groups, including age, weight, height and BMI. The demographics and additional baseline clinical characteristics such as FPG and HbA1c are presented in **Table 1**.

**Table 1:** Demographics of the subjects at baseline.

Characteristics	Cetagliptin		Sitagliptin 100 mg (N = 8)	Placebo (N = 4)
	50 mg (N=10)	100 mg (N=10)		
Age (year)	47.80 ± 4.32	45.20 ± 9.83	50.75 ± 6.25	43.75 ± 18.45
Weight (kg)	69.55 ± 7.63	72.56 ± 7.62	67.70 ± 12.38	68.75 ± 10.19
Height (cm)	164.98 ± 4.71	167.85 ± 9.50	164.61 ± 9.27	163.63 ± 7.97
BMI (kg/m^2^)	25.49 ± 1.88	25.80 ± 2.32	24.79 ± 2.14	25.56 ± 1.69
Gender (male)	90.00%	70.00%	50.00%	50.00%
FPG (mmol/L)	7.96 ± 1.29	6.87 ± 1.40	7.47 ± 1.46	5.90 ± 1.42
HbA1c (%)	8.21 ± 0.66	7.79 ± 0.53	8.01 ± 0.48	7.93 ± 0.90
GA (%)	23.00 ± 3.00	19.98 ± 2.05	22.10 ± 1.92	19.58 ± 3.49

BMI, body mass index; FPG, fasting plasma glucose; GA, glycated Albumin.

### 3.2 Safety and tolerability

A total of 18 subjects experienced 32 AEs, of which 12 AEs were considered to be possibly, probably, or definitely related to the drug. The drug-related AEs included 3 AEs reported by 2 subjects in the cetagliptin group (1 subject experienced hunger feeling, 1 subject experienced diarrhea and upper abdominal discomfort), 7 AEs reported by 3 subjects in the sitagliptin group (1 subject experienced increased white blood cell count, increased neutrophil count, increased lymphocyte count, prolonged QT interval, and elevated level of triglyceride; 1 subject experienced prolonged QT interval; 1 subject experienced elevated level of triglyceride), and 2 AEs reported by 1 subjects in the placebo group (1 subject experienced mouth ulcer and elevated level of triglyceride). All the drug-related AEs were mild in intensity except the mouth ulcer which was moderate, and were resolved at the end of the study. None of the 32 subjects had clinically significant abnormal liver function.

Cetagliptin appeared to be safe and well tolerated, with no serious AEs or withdraws due to AEs throughout the study.

### 3.3 Pharmacokinetic evaluation

**Figure 1:**
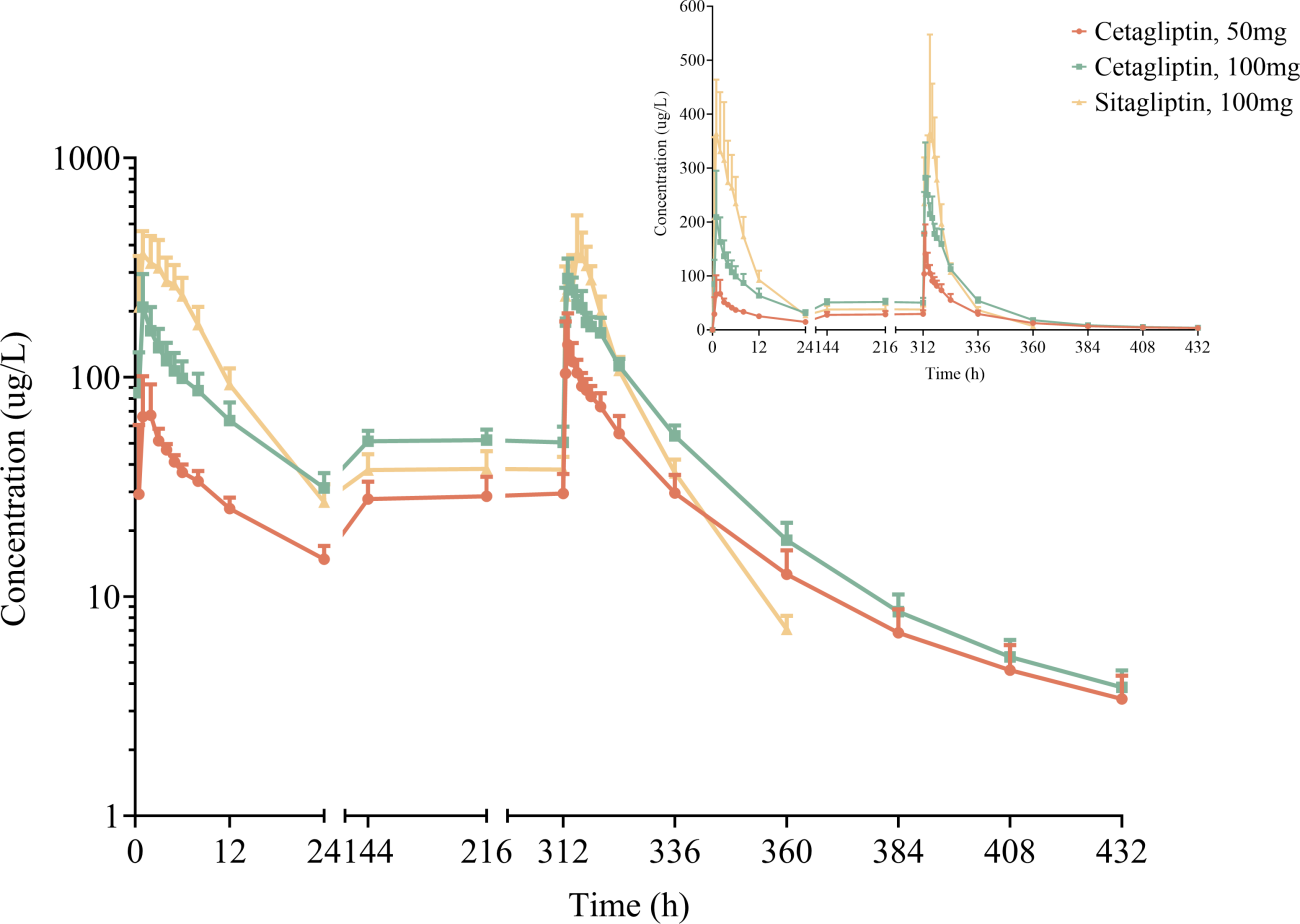
The mean (SD) trough plasma concentration-time profiles after multiple oral doses of cetagliptin and sitagliptin in patients with T2DM.

Following multiple oral doses of cetagliptin 50/100 mg and sitagliptin 100 mg, the mean plasma concentration-time profiles for cetagliptin and sitagliptin are depicted in **Figure 1**, and the main corresponding PK parameters are summarized in **Table 2**. There were no significant differences in plasma trough concentrations between days 7, 10 and 14 (**Figure 1**), suggesting that the steady-state conditions were reached after 1 week of daily dosing.

**Table 2:** Pharmacokinetic parameters after single and multiple oral doses of cetagliptin and sitagliptin in patients with T2DM.

Day	Parameters	Cetagliptin		Sitagliptin 100 mg (N=8)
		50 mg (N=10)	100 mg (N=10)	
Day 1	T_max_ (h)	2.00 (1.00-4.00)	1.00 (1.00-3.00)	1.50 (1.00-5.00)
	C_max_ (ng/mL)	80.5 ± 23.3	219 ± 72.3	394 ± 105
	AUC_0-24h_ (h*ng/mL)	717 ± 86.0	1830 ± 347	3340 ± 522
Day 14	T_max,ss_ (h)	1.00 (0.500-5.00)	1.00 (0.500-3.00)	3.00 (0.500-5.00)
	C_max,ss_ (ng/mL)	162 ± 58.1	300 ± 46.9	419 ± 137
	AUC_0-24h_ (h*ng/mL)	1530 ± 274	3120 ± 263	3760 ± 748
	AUC_0-t_ (h*ng/mL)	2510 ± 512	4580 ± 356	4290 ± 783
	AUC_0-∞_ (h*ng/mL)	2710 ± 577	4780 ± 361	4380 ± 789
	t_1/2_ (h)	41.9 ± 11.0	34.9 ± 12.3	9.12 ± 0.664
	V_z,ss_/F (L)	2010 ± 610	1640 ± 628	362 ± 75.9
	CL_ss_/F (L/h)	33.6 ± 5.86	32.2 ± 2.57	27.5 ± 5.12
	C_avg_ (ng/mL)	63.9 ± 11.4	130 ± 11.0	157 ± 31.2
	R_Cmax_	2.01 ± 0.417	1.49 ± 0.542	1.10 ± 0.121
	R_AUC_	2.13 ± 0.225	1.75 ± 0.305	1.13 ± 0.343

Cetagliptin was rapidly absorbed after administration and the plasma concentrations of cetagliptin peaked between 0.5 and 5 h postdose, and then declined in a biphasic manner with a mean t_1/2_ of 34.9-41.9 h. The CL/F did not change after multiple doses of 50 and 100 mg cetagliptin (33.6 vs 32.2). For 50 mg of cetagliptin, the C_max_ and AUC_0-24h_ values on day 1 were 80.5 ng/mL and 717 h*ng/mL, respectively. The corresponding values on day 14 were 162 ng/mL and 1530 h*ng/mL, respectively. The mean accumulation values for C_max_ and AUC_0-24h_ were 2.01 and 2.13, respectively. For 100 mg of cetagliptin, the C_max_ and AUC_0-24h_ values on day 1 were 219 ng/mL and 1830 h*ng/mL, respectively. The corresponding values on day 14 were 300 ng/mL and 3120 h*ng/mL, respectively. The mean accumulation values for C_max_ and AUC_0-24h_ were 1.49 and 1.75, respectively. These results indicated that there was a modest accumulation of cetagliptin after multiple doses.

Additionally, compared with cetagliptin, sitagliptin showed similar T_max_ and shorter t_1/2_ (9.12 _h_ vs 34.9-41.9 h). The C_max_ and AUC accumulation values revealed no accumulation of sitagliptin after multiple doses.

### 3.4 Pharmacodynamic evaluation

#### 3.4.1 DPP-4 inhibition

**Figure 2:**
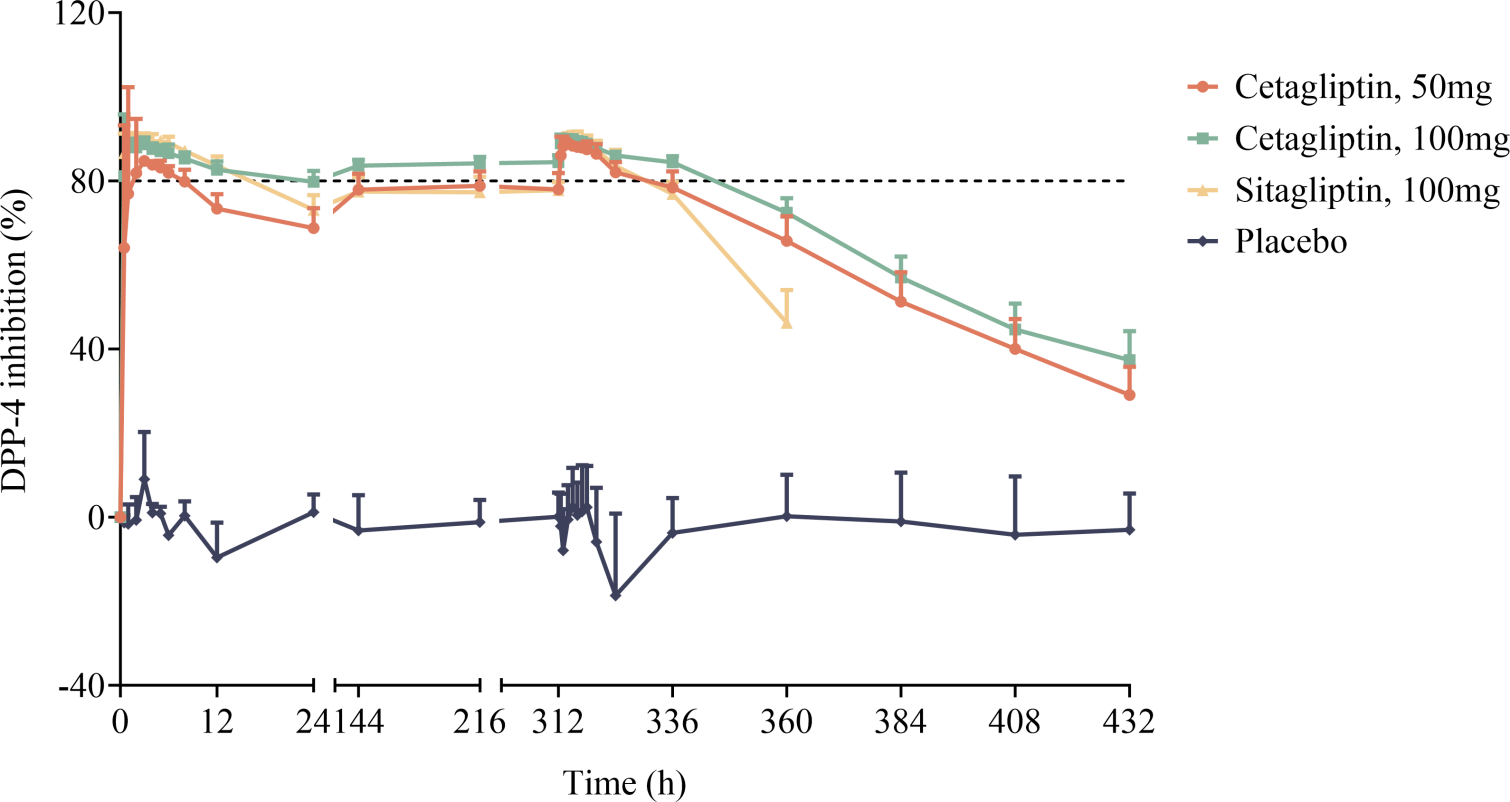
The mean plasma DPP-4 inhibition-time profiles after oral dose administration of cetagliptin, sitagliptin, and placebo in patients with T2DM.

Mean plasma DPP-4 inhibition-time profiles of cetagliptin, sitagliptin, and placebo are shown in **Figure 2**, and the PD parameters are summarized in **Table 3**.

**Table 3:** Pharmacodynamic parameters of DPP-4 inhibition for cetagliptin and sitagliptin in patients with T2DM.

Day	Parameters	Cetagliptin		Sitagliptin 100 mg (N=7)
		50 mg (N=10)	100 mg (N=10)	
Day 1	T_Rmax_ (h)	2.00 (0.500-3.00)	1.00 (1.00-3.00)	3.00 (1.00-5.00)
	R_max_ (%)	86.39 ± 1.75	88.78 ± 1.04	90.10 ± 1.45
	AUEC_0-24h_ (h*%)	1820 ± 77.9	2000 ± 41.6	1970 ± 38.9
	DUR_80%_ (h)	8.39 ± 2.37	21.1 ± 3.42	16.3 ± 2.63
	E_24h_ (%)	68.75 ± 4.75	79.75 ± 2.63	73.11 ± 3.53
Day 14	T_Rmax,ss_ (h)	2.00 (0.500-6.00)	2.00 (1.00-3.00)	4.00 (2.00-5.00)
	R_min,ss_ (%)	77.24 ± 4.52	84.28 ± 1.61	77.21 ± 2.48
	R_max,ss_ (%)	89.47 ± 1.12	89.99 ± 0.91	90.43 ± 1.51
	AUEC_0-24h_ (h*%)	2010 ± 54.1	2090 ± 24.4	2000 ± 74.6
	DUR_80%_ (h)	21.9 ± 7.05	32.3 ± 4.53	18.6 ± 4.94
	E_24h_ (%)	78.43 ± 3.85	83.91 ± 1.39	77.72 ± 3.02

As shown in **Figure 2**, compared with the placebo group, plasma DPP-4 activity was significantly inhibited following administration of cetagliptin or sitagliptin. After single administration, the R_max_ values for 50 mg cetagliptin, 100 mg cetagliptin, and sitagliptin were 86.39, 88.78, and 90.10%, respectively. The corresponding DUR_80%_ values were 8.39, 21.1, and 16.3 h, respectively. The results showed that the intensity of DPP-4 inhibition induced by 100 mg cetagliptin was comparable with that induced by sitagliptin, while the duration of inhibition was longer than that of sitagliptin. The DPP-4 inhibition reached a steady state (**Figure 2**) after 1 week of daily dosing. After multiple administration, the R_max_ values for 50 mg cetagliptin, 100 mg cetagliptin, and sitagliptin were 89.47, 89.99, and 90.43%, respectively. The corresponding DUR_80%_ values were 21.9, 32.3, and 18.6 h, respectively. And the E_24h_ were 78.43, 83.91, and 77.72%, respectively, suggesting that the intensity and duration of DPP-4 inhibition induced by 50 mg cetagliptin was comparable with that induced by sitagliptin, and 100 mg cetagliptin showed a much longer sustained DPP-4 inhibition (≥80%) than sitagliptin. Meanwhile, the accumulation ratios of AUEC_0-24h_ for 50 mg cetagliptin, 100 mg cetagliptin, and sitagliptin were close to 1.

**Figure 3:**
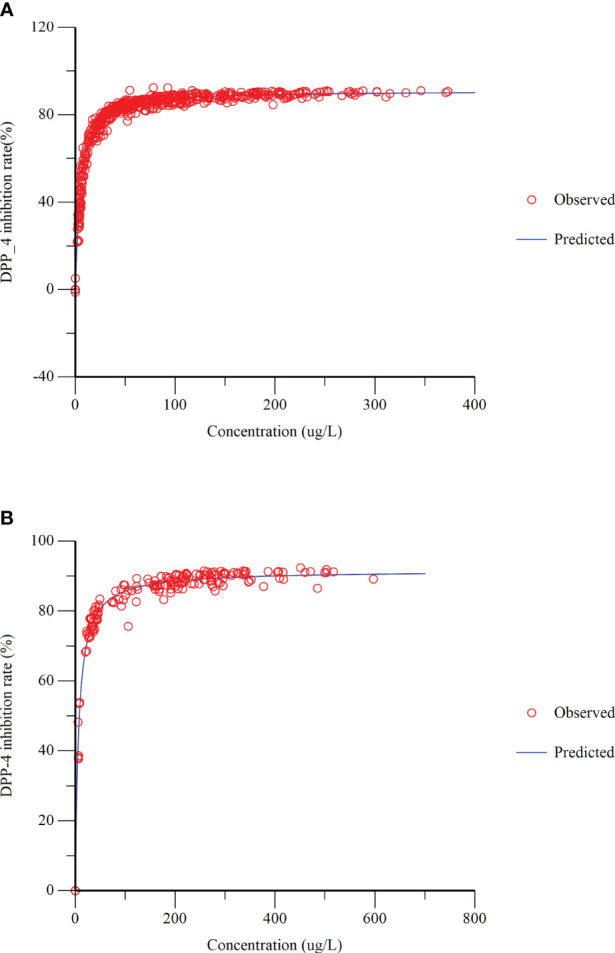
Dipeptidyl peptidase-4 (DPP-4) inhibition-concentration Emax model fitting. **(A)** cetagliptin; **(B)** sitagliptin.

The relationship between plasma concentrations of cetagliptin or sitagliptin and DPP-4 inhibition was evaluated by an E_max_ model (**Figure 3**). The DPP-4 inhibitory intensity increased with the drug concentration and reached a plateau. The E_max_ values for cetagliptin and sitagliptin were 92.47% and 91.68%, respectively. And EC_50_ values were 5.37 and 6.73 ng/mL, respectively.

#### 3.4.2 Active GLP-1 concentrations

**Figure 4:**
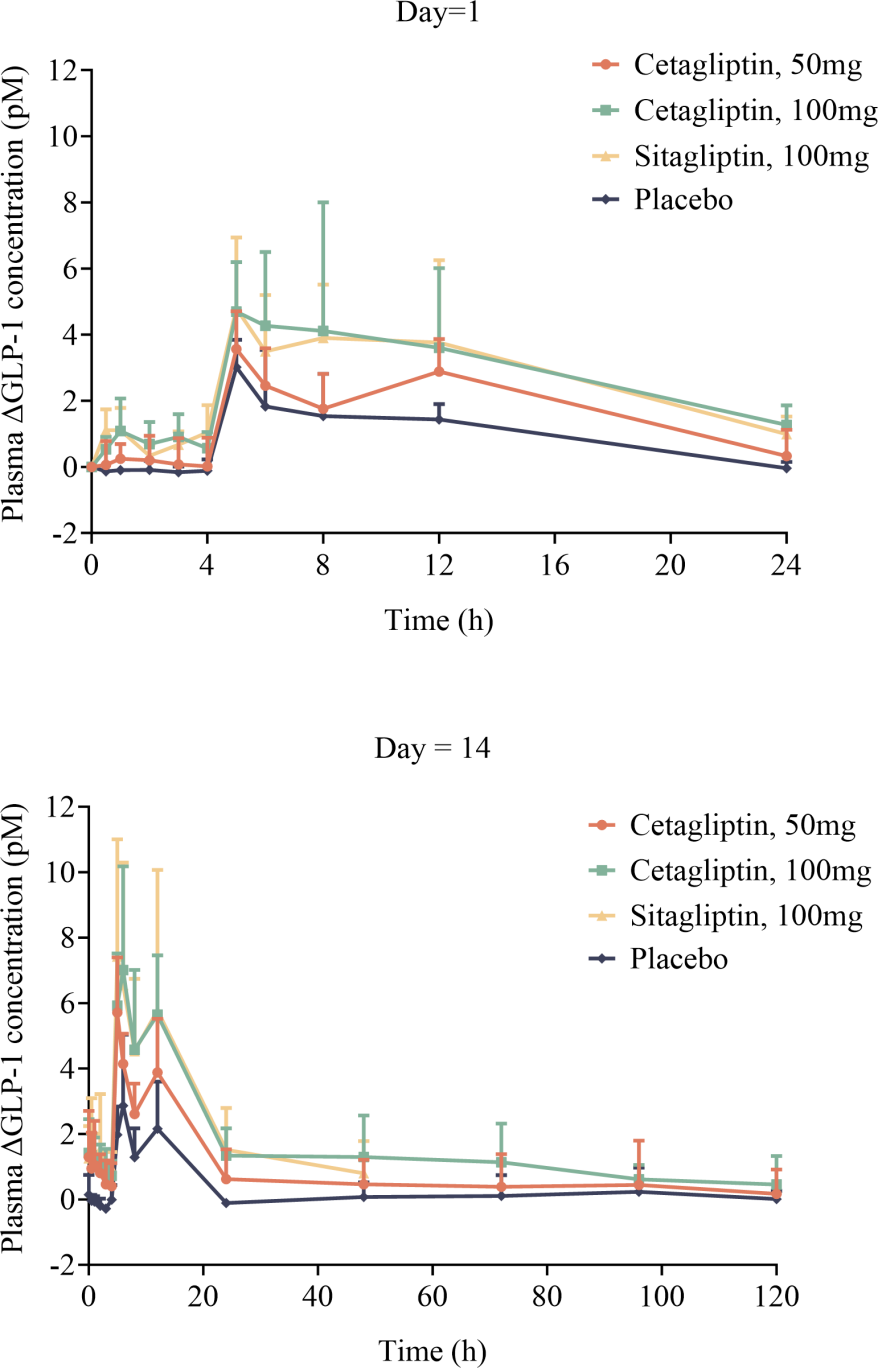
The mean plasma △GLP-1 –time curves of cetagliptin, sitagliptin, or placebo after single and multiple oral doses.

As shown in **Figure 4**, the T_Rmax_ of plasma active GLP-1 in cetagliptin, sitagliptin and placebo groups were similar. Plasma active GLP-1 concentrations were influenced by diet and increased after meals at 4 h and 10 h post dose. While compared with the placebo group, plasma active GLP-1 concentrations were much higher in cetagliptin and sitagliptin groups.

**Table 4** shows the PD parameters of active GLP-1 following administration of cetagliptin, sitagliptin, and placebo in patients with T2DM. After single administration, the main parameters such as R_max_, AUEC_0-24h_, △GLP-1-6h and △GLP-1-12h in 100 mg cetagliptin group were higher than those in 50 mg cetagliptin group and comparable with those in 100 mg sitagliptin group (except for △GLP-1-6h, the former group was higher).

**Table 4:** A summary of △GLP-1 pharmacodynamic parameters of cetagliptin, sitagliptin, and placebo.

Day	Parameters	Cetagliptin		Sitagliptin 100 mg (N=8)	Placebo (N=4)
		50 mg (N=10)	100 mg (N=10)		
Day 1	Baseline (pM)	0.491 ± 0.815	0.434 ± 0.393	0.442 ± 0.406	0.282 ± 0.131
	T_Rmax_ (h)	5.00 (5.00-12.00)	5.00 (5.00-12.00)	7.00 (5.00-12.00)	5.00 (5.00-6.00)
	R_max_ (pM)	3.866 ± 1.031	6.026 ± 3.823	5.901 ± 2.375	3.131 ± 0.976
	AUEC_0-24h_ (h*pM)	42.6 ± 10.0	63.1 ± 34.3	61.2 ± 28.6	21.1 ± 11.4
	△GLP-1-6h (pM)	2.457 ± 1.135	4.269 ± 2.232	3.491 ± 1.703	1.828 ± 1.711
	△GLP-1-12h (pM)	2.887 ± 0.981	3.600 ± 2.410	3.764 ± 2.490	1.438 ± 0.460
Day 14	R_0h,ss_ (pM)	1.787 ± 1.046	1.858 ± 1.186	1.700 ± 0.851	0.428 ± 0.593
	T_Rmax,ss_ (h)	5.00 (5.00-12.00)	6.00 (5.00-12.00)	5.00 (5.00-6.00)	6.00 (6.00-12.00)
	R_max,ss_ (pM)	5.891 ± 1.692	7.868 ± 2.565	8.080 ± 3.592	3.511 ± 1.959
	R_avg,ss_ (pM)	2.408 ± 0.944	3.673 ± 1.234	3.821 ± 2.361	1.094 ± 0.648
	AUEC_0-24h_ (h*pM)	57.8 ± 22.7	88.2 ± 29.6	91.7 ± 56.7	26.3 ± 15.5
	△GLP-1-6h (pM)	4.145 ± 0.920	7.006 ± 3.171	6.553 ± 3.739	2.864 ± 2.174
	△GLP-1-12h (pM)	3.880 ± 1.641	5.648 ± 1.810	5.775 ± 4.299	2.161 ± 1.442

After multiple administration for 14 days, steady-state conditions were achieved. The baseline plasma active GLP-1 concentrations (R_0h_) on day 14 in 50 mg cetagliptin, 100 mg cetagliptin, and sitagliptin groups were similar (1.787 vs 1.858 vs 1.700 pM). And the comparison results of the main parameters (such as R_max_, AUEC_0-24h_, △GLP-1-6h and △GLP-1-12h) in each group after multiple administration were consistent with those after single administration.

#### 3.4.3 Effects on glucose, insulin, C-peptide, and glucagon

Compared with baseline, after administration of cetagliptin or sitagliptin, plasma glucose and glucagon levels showed an obvious decrease, while insulin and C-peptide showed an obvious increase (**Supplementary Figure 1**).

Meanwhile, the changes of AUEC_0-3 h_ relative to baseline for plasma glucose in 50 mg cetagliptin, 100 mg cetagliptin, sitagliptin, and placebo were -4.97, -2.76, -0.66, and 1.61 h*mmol/L, respectively. The corresponding changes for insulin were 219.90, 292.18, 115.99, and 82.68 h*mmol/L, respectively. Changes for C-peptide were 1851.30, 1761.48, 1046.88, and 334.33 h*mmol/L, respectively. Changes for glucagon were -72.70, -7.21, -26.29, and 32.17 h*mmol/L, respectively (**Table 5**). These results indicated that a trend of decline in plasma glucose and a trend of improvement of pancreatic β-cell function were observed after administration of cetagliptin.

**Table 5:** Pharmacodynamic parameters for OGTT on day -1 and day 15.

Day	PD index	Parameters	Cetagliptin		Sitagliptin 100 mg (N=8)	Placebo (N=4)
			50 mg (N=10)	100 mg (N=10)		
Day -1	glucose	AUEC_0-t_ (h*mmol/L)	48.65 ± 5.34	40.59 ± 2.87	44.07 ± 5.74	42.51 ± 2.82
	insulin	AUEC_0-t_ (h*mmol/L)	431.53 ± 271.81	716.44 ± 250.84	769.99 ± 616.93	889.74 ± 293.88
	C-peptide	AUEC_0-t_ (h*mmol/L)	4072.02 ± 1174.82	6059.64 ± 1263.61	5481.21 ± 2115.5	6261.57 ± 1421
	glucagon	AUEC_0-t_ (h*mmol/L)	469.51 ± 189.76	503.79 ± 161.61	456.48 ± 142.02	448.09 ± 1.82
Day 15	glucose	AUEC_0-t_ (h*mmol/L)	43.68 ± 8.04	37.83 ± 5.09	43.40 ± 6.51	44.12 ± 9.69
	insulin	AUEC_0-t_ (h*mmol/L)	651.43 ± 455.05	1008.62 ± 367.20	885.98 ± 443.33	972.42 ± 282.79
	C-peptide	AUEC_0-t_ (h*mmol/L)	5923.31 ± 2012.97	7821.13 ± 2023.09	6528.08 ± 1123.8	6595.90 ± 1447
	glucagon	AUEC_0-t_ (h*mmol/L)	396.81 ± 51.89	496.58 ± 158.93	430.19 ± 63.90	480.26 ± 61.42

### 3.5 Preliminary efficacy evaluation

The changes of FPG relative to baseline (day -2) on day 7 in 50 mg cetagliptin, 100 mg cetagliptin, sitagliptin, and placebo were 0.49, -0.11, 0.04, and 0.40 mmol/L, respectively. And the corresponding changes on day 14 were 0.56, 0.43, 0.68, and 1.91 mmol/L, respectively. The results showed that no obvious FPG-lowering effect was observed after administration of cetagliptin or sitagliptin. Compared with baseline, the 2 h PPG values on day 7 and day 14 decreased in the 50 mg cetagliptin group, particularly the 2 h PPG on day 14 after dinner which decreased by 2.64 mmol/L (**Table 6**).

**Table 6:** The mean change value of 2h postprandial blood glucose concentration in day 7 and day 14 after medication compared with baseline.

Day	Time	Cetagliptin		Sitagliptin 100 mg (N=8)	Placebo (N=4)
		50 mg (N=10)	100 mg (N=10)		
Day 7	2 h after breakfast	-1.54 ± 2.25	-0.54 ± 2.47	-1.04 ± 1.77	-0.15 ± 2.65
	2 h after lunch	-0.29 ± 2.34	-0.61 ± 2.16	0.06 ± 1.87	1.55 ± 3.87
	2 h after supper	-1.30 ± 2.50	1.64 ± 3.22	1.28 ± 3.33	2.13 ± 5.73
Day 14	2 h after lunch	-0.66 ± 2.26	1.16 ± 2.05	0.92 ± 2.60	3.05 ± 3.45
	2 h after supper	-2.64 ± 2.07	2.56 ± 2.48	1.39 ± 2.70	2.17 ± 7.67

Compared with baseline, the HbA1c values in 50 mg cetagliptin, 100 mg cetagliptin, sitagliptin, and placebo decreased by 0.47%, 0.35%, 0.44%, and 0.52%, respectively. The results of one-way ANOVA analysis showed that there was no difference among these treatment groups (P>0.05). Additionally, the changes of GA relative to baseline (day -2) on day 14 in 50 mg cetagliptin, 100 mg cetagliptin, sitagliptin, and placebo were 0.53, -1.70, -1.49, and -0.52%, respectively, indicating that GA tended to decrease after administration of 100 mg cetagliptin or sitagliptin.

### 3.6 Population PKPD analysis

#### 3.6.1 Final population PK model

The two-compartmental model was chosen as the structural model. An exponential variability error model was used to describe inter-individual variability, and a proportional error model was selected to account for residual variability. Covariate searches with the stepwise method identified TBIL as significant effect on V2, and covariate TBIL was included in the final population PK model. After including the covariates, the -2LL value of the model decreased from 4335 to 4326. (Δ-2LL=9). The final model parameters are summarized in **Supplementary Table 1**. The Goodness-of-Fit (GOF) plots of the final model are shown in **Supplementary Figures 2B, C**. The GOF plots showed that the final model fitted most of the observed data well, but there were individual data deviations. The plots for CWRES vs. Time or population predictions are shown in **Supplementary Figure 2A**. Most CWRES were symmetrically distributed on both sides of the line (y=0) without significant deviation.

The resampling process was repeated 500 times by bootstrapping, and the median parameter values and 95% confidence interval (95% CI) results are summarized in **Supplementary Table 1**. The median values were similar to those estimated by the final model, and the parameter estimates from the original data were all within 95% CI. Therefore, the final model has good stability.

**Figure 5:**
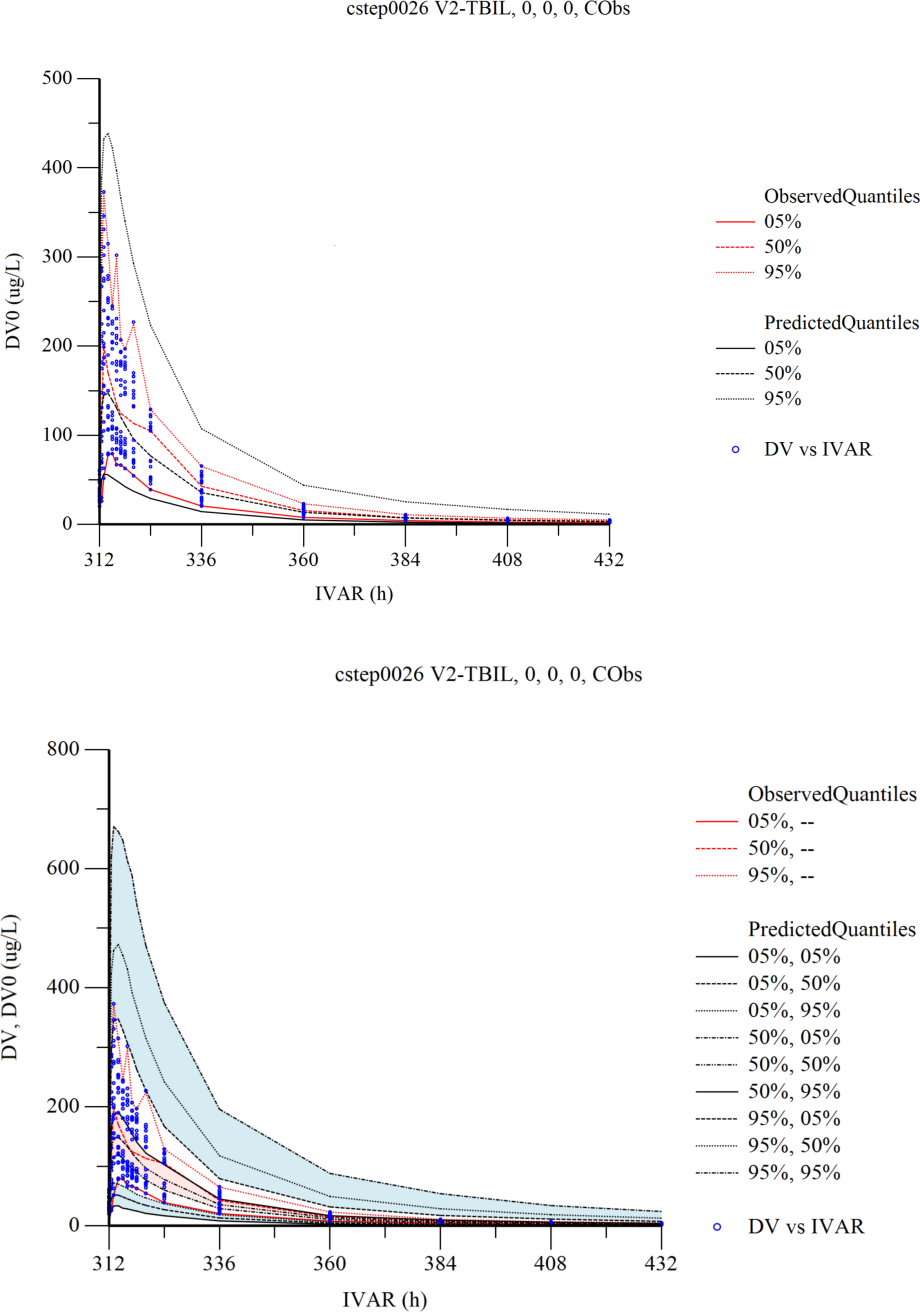
Visual predictive check (VPC) from the final population pharmacokinetic model. Red solid and dashed lines represent the 5 th, 50 th, and 90 th percentiles of the observed concentrations. the 3 shaded areas represent the 90% CIs of the simulated concentrations’ 5th, 50th, and 95th percentiles. The dots represent the observed data. DV, observed concentration; IVAR, Time.

The VPC results are shown in **Figure 5**. In the VPC plots, the 90% prediction interval (90% PI) is the region between the predicted 5th and 95th percentiles. Most of the observations fell within 90% PI. The 5th, 50th and 95th quantiles of the observed values showed a similar trend to the 5th, 50th and 95th quantiles of the predicted values. The figure indicates that the final model has sufficient predictive power.

#### 3.6.2 Final population PK/PD model

The population PK/PD model of cetagliptin was established using the Sigmoid-E_max_ model. The mixed error model illustrated the residual variability. The stepwise method was used for covariate screening, and no covariates were found to significantly affect PK/PD parameters. The estimates, relative standard errors (RSE), and inter-individual variation of the final model parameters are summarized in **Supplementary Table 1**. The GOF plots of the final population PK/PD model are shown in **Supplementary Figures 3B, C**. The results showed that the final model fitted the observed data well without significant deviation. The plots for CWRES vs. Time or population predictions are shown in **Supplementary Figure 3A**. Most of the CWRES were distributed between ±4, but the CWRES showed obvious trend changes, suggesting that the model needs further optimization.

The resampling process was repeated 500 times by bootstrapping, and the median parameter values and 95% confidence interval (95% CI) results are summarized in **Supplementary Table 1**.The median values were similar to the parameter values estimated by the final model, and those estimated by the model were all within 95% CI. As a result, the final model has good stability.

**Figure 6:**
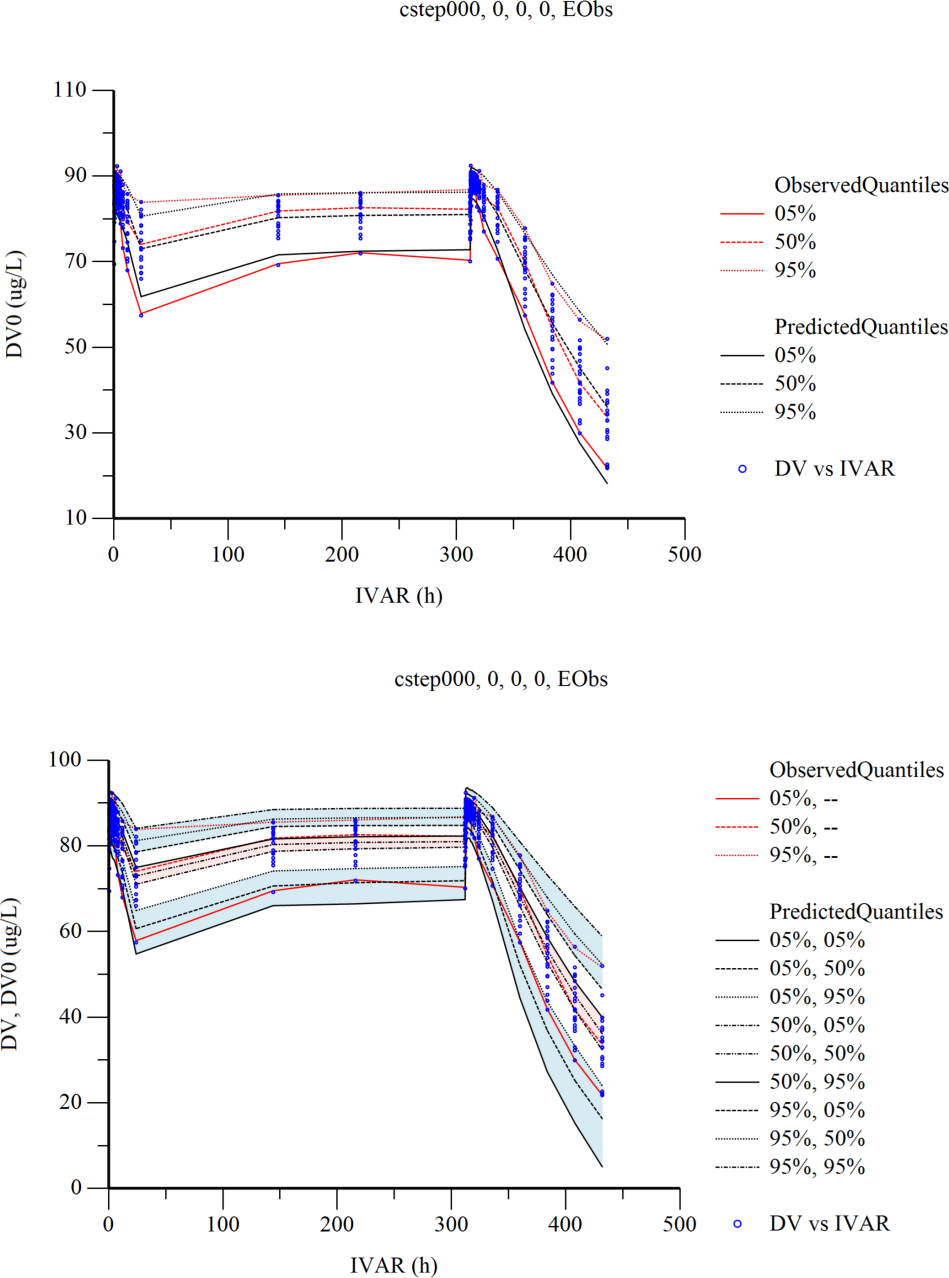
Visual predictive check (VPC) from the final population pharmacokinetic/pharmacodynamic model. Red solid and dashed lines represent the 5 th, 50 th, and 90 th percentiles of the observed concentrations. the 3 shaded areas represent the 90% CIs of the simulated concentrations’ 5th, 50th, and 95th percentiles. The dots represent the observed data. DV, observed concentration; IVAR, Time.

The Visual Predictive Check (VPC) results are shown in **Figure 6**. In the VPC plots, the 90% prediction interval (90% PI) is the region between the predicted 5th and 95th percentiles. Most of the observations fell within 90% PI. The 5th, 50th and 95th quantiles of the observed values showed a similar trend to the 5th, 50th and 95th quantiles of the predicted values. The figure shows that the final model has adequate predictive capability.

## 4 Discussion

This study evaluated the safety, PK, and PD of cetagliptin in Chinese patients with T2DM, using sitagliptin as a positive control. In therapeutic doses, oral administration of cetagliptin (50 or 100 mg) or sitagliptin (100 mg) was well tolerated and safe. All AEs appeared in cetagliptin and sitagliptin groups were mild, the AEs of cetagliptin were similar to those listed in the label of sitagliptin, and there was no new safety signal. No serious adverse events occurred in any treatment groups, and no AEs led to discontinuation of the trial.

After 1 week of daily dosing, the plasma concentrations of cetagliptin or sitagliptin reached a steady-state. The steady-state’s primary PK parameters of 50 or 100 mg cetagliptin in patients with T2DM were similar with those in the healthy subjects (31), with C_max_ of 162 vs 125 ng/mL, T_max_ of 1.0 vs 1.5 h, AUC_0-τ_ of 1530 vs 1440 h*ng/mL, t_1/2_ of 41.9 vs 38.8 h, and R_AUC_ of 2.13 vs 1.72 for 50 mg of cetagliptin; with C_max_ of 300 vs 294 ng/mL, T_max_ of 1.0 vs 1.0 h, AUC_0-τ_ of 3120 vs 3120 h*ng/mL, t_1/2_ of 34.9 vs 36.6 h, and R_AUC_ of 1.75 vs 1.38 for 100 mg of cetagliptin. Meanwhile, the aforementioned main PK parameters of sitagliptin in patients with T2DM were also similar with those in the healthy subjects (31). Compared with sitagliptin, cetagliptin exhibits a much longer elimination half-life (41.9 h in 50 mg cetagliptin, 34.9 h in 100 mg cetagliptin vs 9.12 h in sitagliptin group), indicating that cetagliptin may have longer effect time than sitagliptin and supporting a once-daily dosing regimen of cetagliptin in the following phase II and III clinical studies.

Plasma DPP-4 activity was significantly inhibited after administration of cetagliptin or sitagliptin. The steady-state’s PD parameters (such as R_max_, DUR_80_, E_24h_) for DPP-4 inhibition of cetagliptin and sitagliptin in patients with T2DM were consistent with those in healthy subjects (31). At steady-state, the intensity and duration of DPP-4 inhibition induced by 50 mg cetagliptin was comparable with that induced by sitagliptin, and 100 mg cetagliptin showed a much longer sustained DPP-4 inhibition (≥80%) than sitagliptin. The DPP-4 inhibitory intensity increased with the drug concentrations, and finally reached a “ceiling”. The E_max_ model results showed that the E_max_ for cetagliptin and sitagliptin were 92.47% and 91.68%, respectively, and EC_50_ values were 5.37 and 6.73 ng/mL, respectively, which were also in line with the healthy subjects (31). The results suggested that there was no significant difference in the DPP-4 inhibition in patients with T2DM and healthy subjects following administration of cetagliptin or sitagliptin. When compared with the placebo treatment group, plasma active GLP-1 concentrations were much higher in cetagliptin and sitagliptin groups, and the AUEC_0-24h_ of plasma active GLP-1 after multiple dosing in the 50 mg cetagliptin, 100 cetagliptin, and sitagliptin groups increased by 2.20-, 3.36- and 2.90-fold, respectively. After single dosing of cetagliptin and sitagliptin, the plasma active GLP-1 PD parameters in patients with T2DM were similar with those in healthy subjects. While, the corresponding PD parameters in patients with T2DM after multiple dosing were better than those in healthy subjects (31). The accumulation ratios of R_max_ and AUEC_0-24 h_ for plasma active GLP-1 in 50 mg cetagliptin, 100 mg cetagliptin, and sitagliptin groups were about 1.5 in patients with T2DM, and about 1.0 in healthy subjects, indicating that patients with T2DM were more sensitive to drugs.

Following OGTT in cetagliptin groups on day 15, the AUEC_0-3h_ values showed an obvious decrease for plasma glucose and glucagon, and an increase for insulin and C-peptide. These results indicated that cetagliptin showed a trend of decline in plasma glucose and a trend of improvement of pancreatic β-cell function. The preliminary efficacy evaluation results showed that no obvious FPG-lowering effect was observed after administration of cetagliptin or sitagliptin, which may be related to the shorter administration time or more significant effect of DPP-4 inhibitors on 2 h PPG than FPG, or due to the small sample size or slight differences of baseline FPG values among subjects. Further research should be conducted in long-term dosing studies. In addition, GA reflects average glucose levels over a much shorter period of time than HbA1c, usually about 2 to 3 weeks (32). After 14 days of dosing, a tendency of reduced GA was observed, whereas no decreasing trend was observed in HbA1c. The efficacy and safety of cetagliptin will be further confirmed in phase III confirmatory clinical study.

Moreover, this study developed a population PK/PD model using a sequential fitting approach. In the process of establishing the population PK model, we investigated the effects of gender, body weight, glutamic-pyruvic transaminase, total bilirubin, triglyceride, low-density lipoprotein cholesterol, glucose, urea, and creatinine on pharmacokinetic parameters, which finally proved that only TBIL had a significant effect on V_2_. There is a certain correlation between TBIL and V_2_, and V_2_ increases with the increase of TBIL. Four observations deviated significantly in the GOF plot of the final population PK/PD model. The blood concentrations at these four points were 7.78µg/L, 2.29µg/L, 4.21µg/L and 3.64µg/L, respectively. By observing the raw data, it can be seen that the measured values of these points are lower than the average concentration at this point, so the above points can be seen to deviate from the Y=X standard line on the DV and IPRED curves. The 2022 *Population Pharmacokinetics Guidance for Industry* issued by the US Food and Drug Administration (FDA) states that individual data points with suspected outliers can be eliminated during model development. The guideline states that in some cases, data points with weighted residuals greater than 5 can be considered outliers (33). Therefore, in establishing the population PK/PD model, 6 CWRES with absolute value greater than 5 were excluded, and the final population PK/PD model included 554 blood concentrations. This model will be used to evaluate the exposure-response relationship of cetagliptin in patients with T2DM, providing valuable guidance for following clinical medication. With the accumulation of new clinical trial data, it is necessary to continuously integrate new data to update and improve the model.

## 5 Conclusion

In conclusion, Chinese patients with T2DM treated with 50 mg or 100 mg of cetagliptin for 14 days showed favorable PK/PD characteristics, safety and tolerance, with a high DPP-4 inhibition rate and a certain trend of glucose-lowering. In addition, the pharmacokinetic profile and exposure-response relationship of cetagliptin in Chinese patients with T2DM after single and multiple doses were quantitatively described. Ultimately, we hope these clinical data and the developed model will inform further studies and guide the dose selection of cetagliptin.

## Data availability statement

The data analyzed in this study is subject to the following licenses/restrictions: Data will be made available on request. Requests to access these datasets should be directed to Feng Shao, jsphshaofeng@hotmail.com.

## Ethics statement

The studies involving humans were approved by Ethics Committee of the First Affiliated Hospital of Nanjing Medical University. The studies were conducted in accordance with the local legislation and institutional requirements. The participants provided their written informed consent to participate in this study.

## Author contributions

CZ: Investigation, Project administration, Writing – review & editing. SZ: Data curation, Formal analysis, Investigation, Project administration, Writing – original draft, Writing – review & editing. JW: Data curation, Writing – original draft. LX: Formal analysis, Project administration, Writing – review & editing. ZL: Writing – original draft. YZ: Data curation, Writing – review & editing. LW: Formal analysis, Methodology, Project administration, Writing – review & editing. HL: Funding acquisition, Writing – review & editing. DX: Funding acquisition, Writing – review & editing. FS: Conceptualization, Methodology, Project administration, Supervision, Writing – review & editing.
