# Supplementary material for: Safety, tolerability, pharmacokinetics and pharmacokinetic-pharmacodynamic modeling of cetagliptin in patients with type 2 diabetes mellitus
Source: Front Endocrinol (Lausanne). 2024 Mar 11;15:1359407. doi: 10.3389/fendo.2024.1359407 (PMC10961402; doi:10.3389/fendo.2024.1359407)
Supplement: Supplementary file 1 [file DataSheet_1.docx]

# Supplementary Figures and Tables

## Supplementary Figures

**Supplementary Figure 1.** The mean plasma glucose, insulin, C-peptide, and glucagon concentration-time profiles after oral dose administration of cetagliptin, sitagliptin, and placebo in patients with T2DM. (A) Glucose, (B) Insulin, (C) C-peptide, (D) Glucagon.


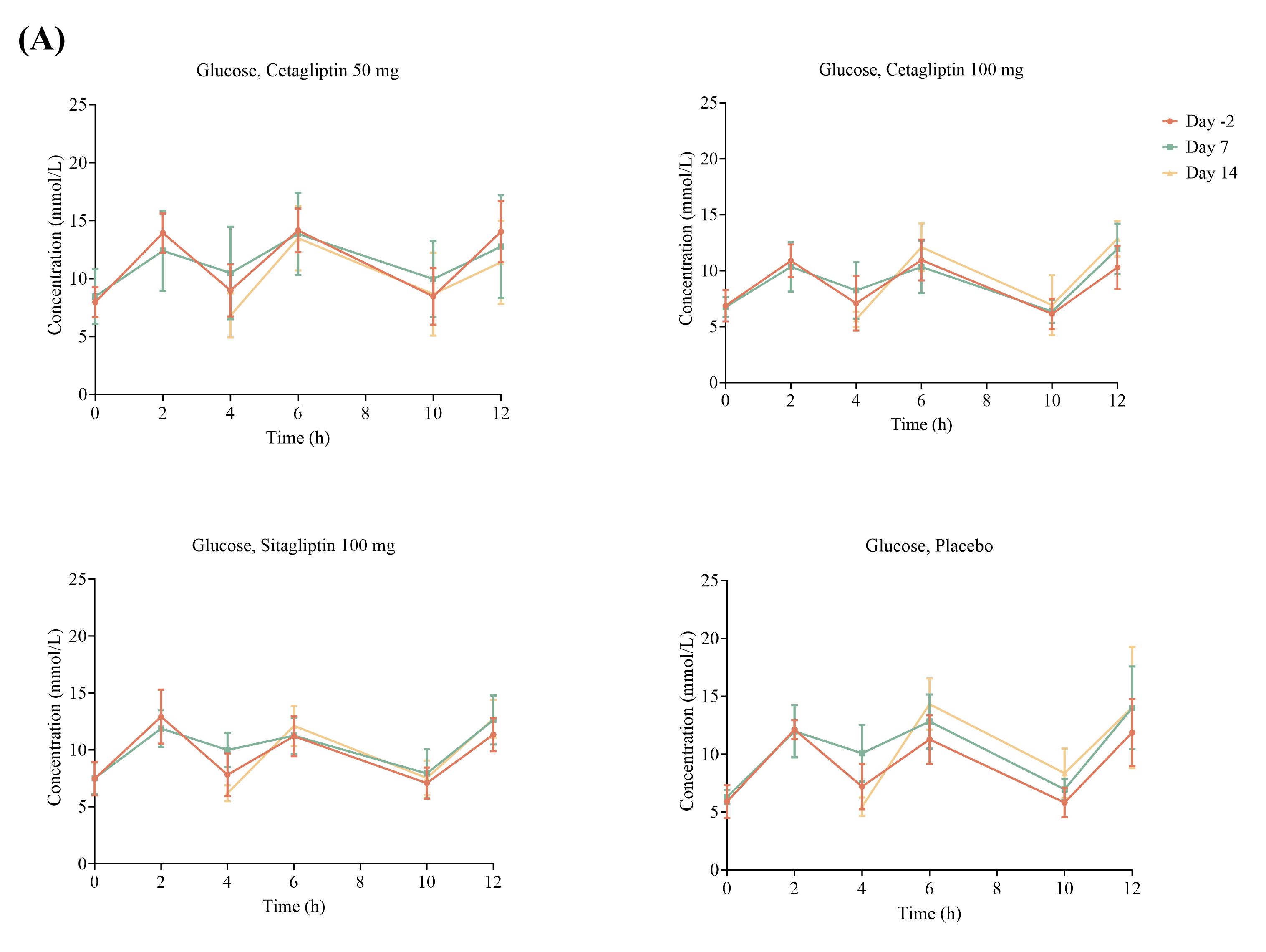


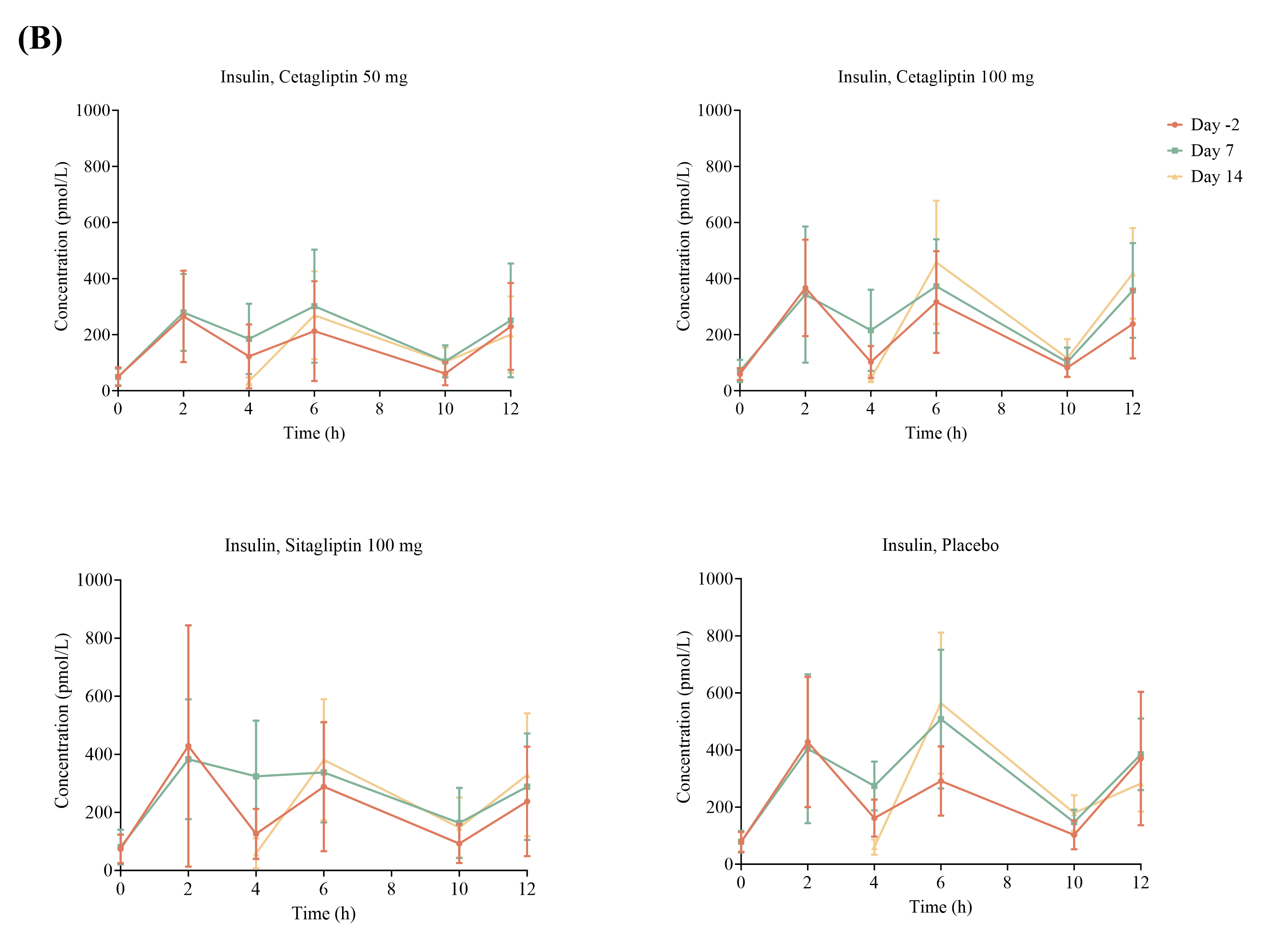

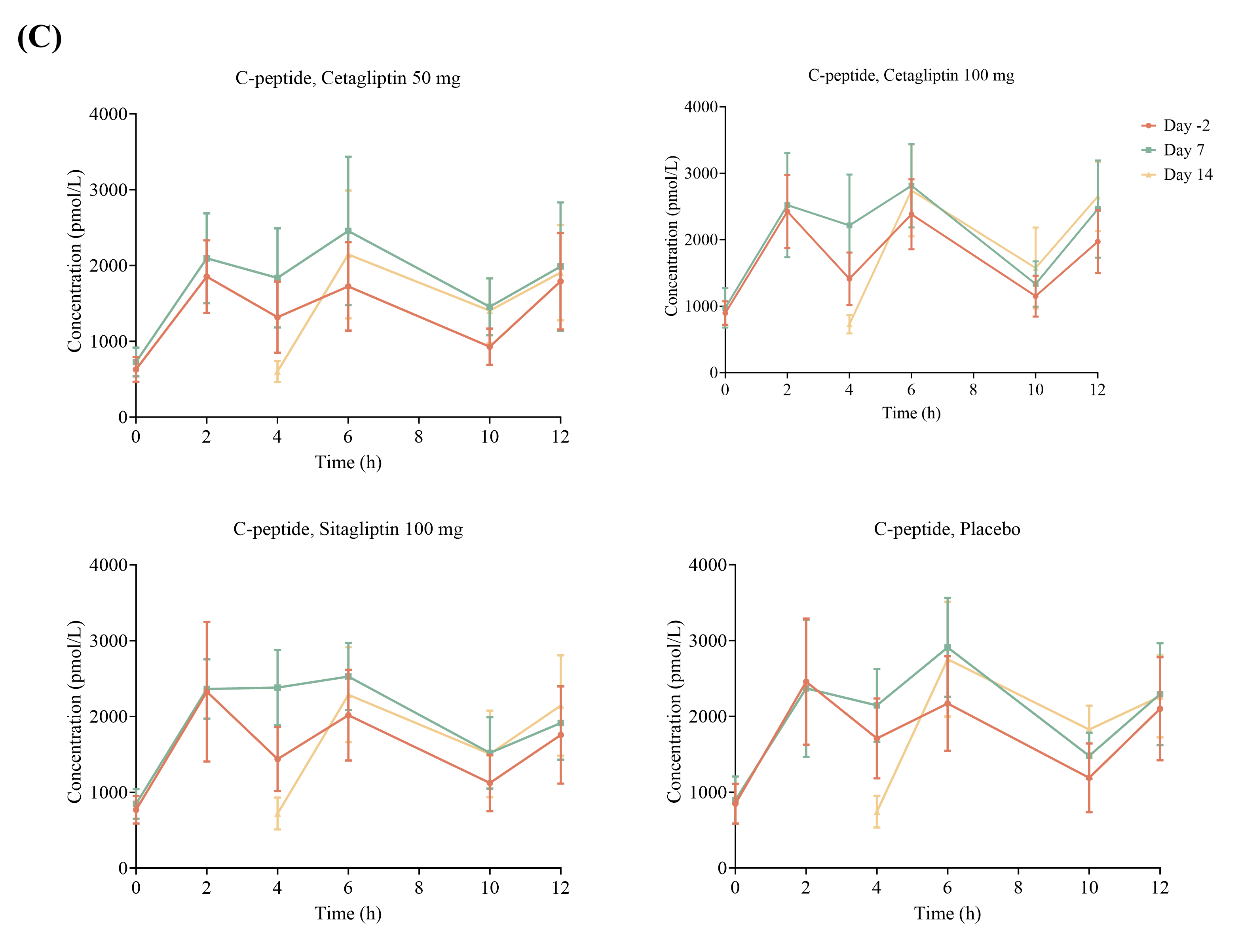


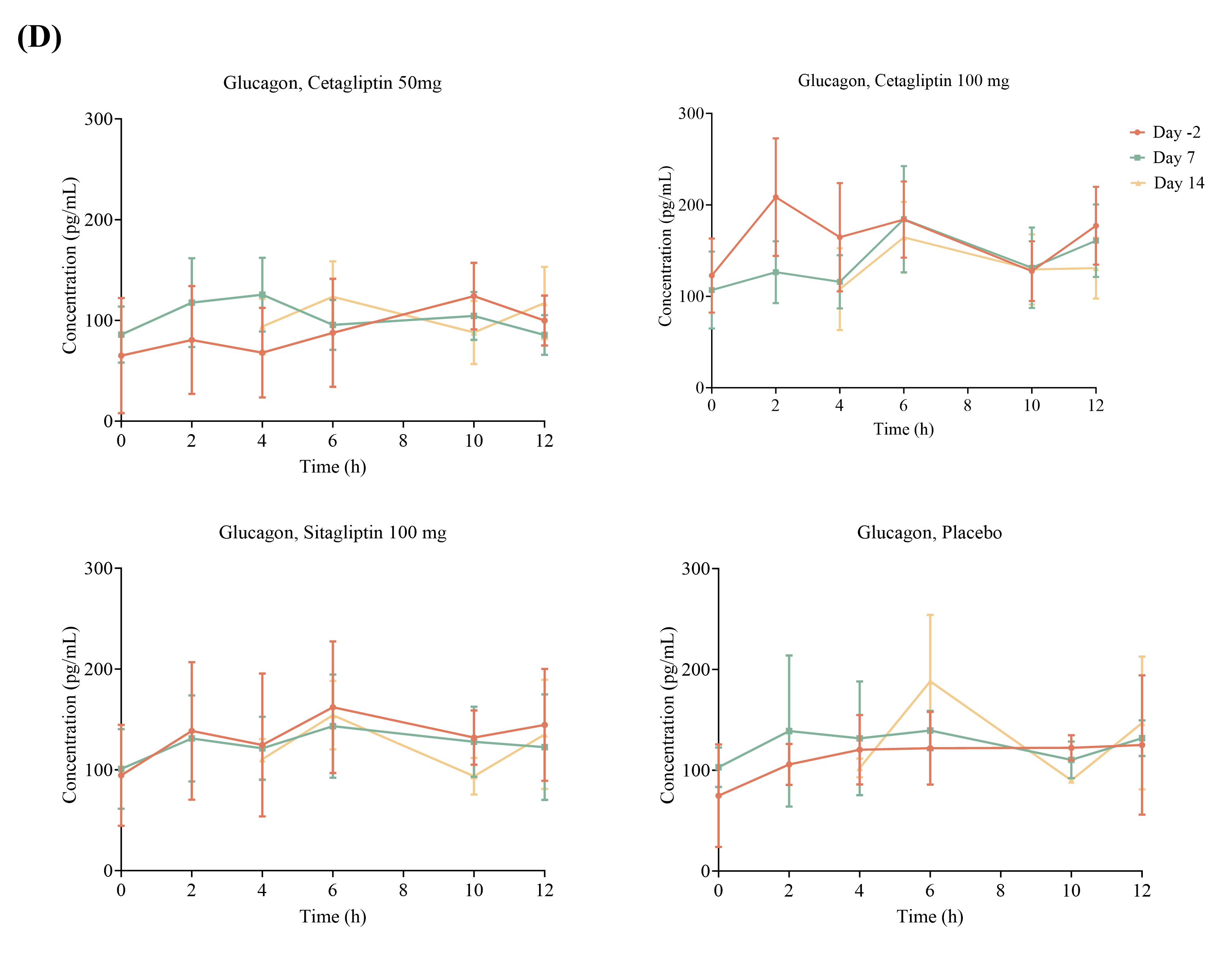


**Supplementary Figure 2.** The goodness-of-fit plots for the final pharmacokinetic model. (A)The left shows that conditional weighted residuals (CWRES) vs. IVAR (time) and the right shows that CWRES vs. Population predictions, the blue solid line is the linear regression trend line; (B) The left shows that observations vs. individual predictions (IPRED) and the right shows that observations vs. individual predictions logarithm, the black solid line is the line of unity y=x; (C)The left shows that observations vs. population predictions (PRED) and the right shows that observations vs. population predictions logarithm, the black solid line is the line of unity y=x.


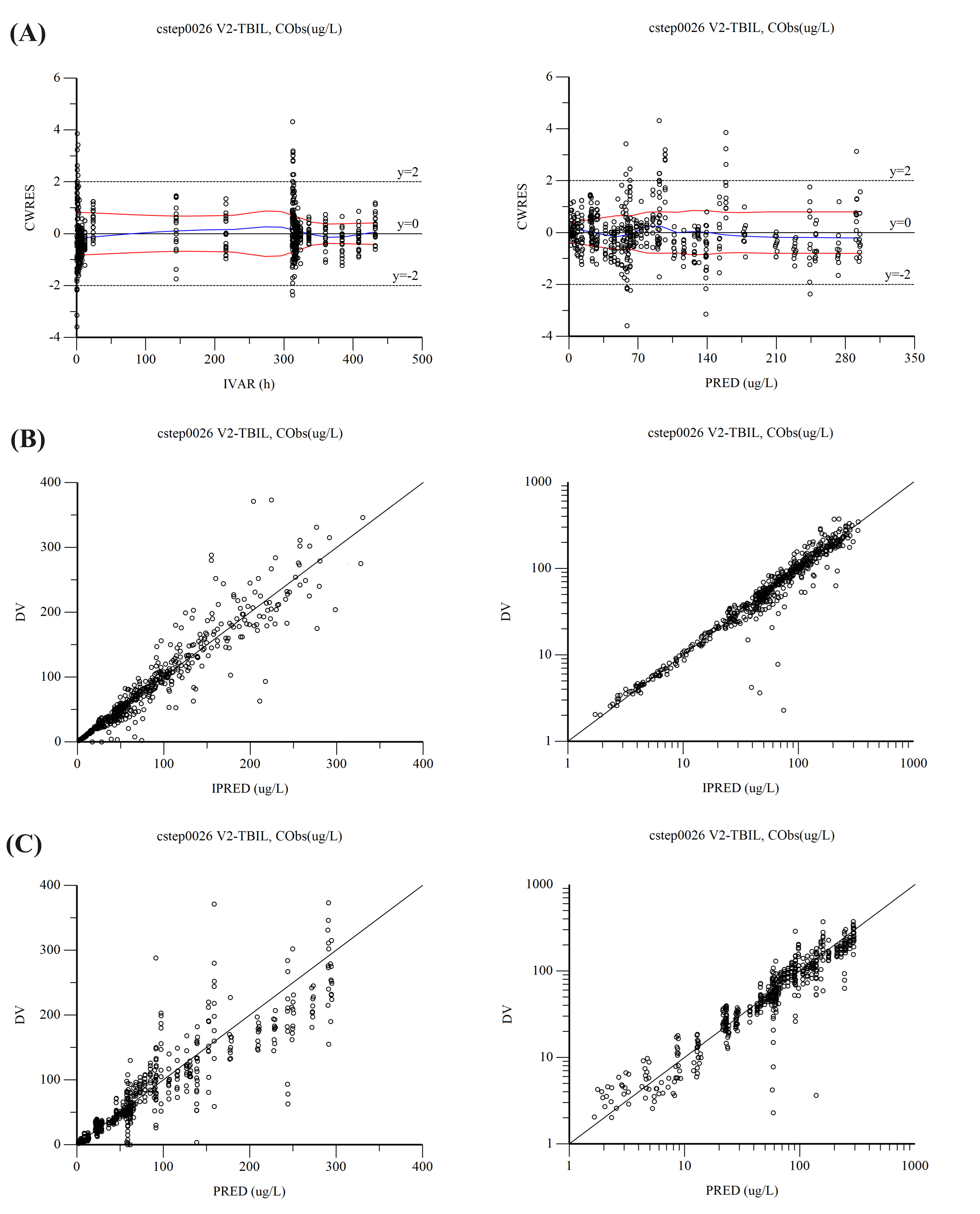


**Supplementary Figure 3.** The goodness-of-fit plots for the final pharmacokinetic/pharmacodynamic model. (A)The left shows that conditional weighted residuals (CWRES) vs. IVAR (time) and the right shows that CWRES vs. Population predictions, the blue solid line is the linear regression trend line; (B) The left shows that observations vs. individual predictions (IPRED) and the right shows that observations vs. individual predictions logarithm, the black solid line is the line of unity y=x; (C)The left shows that observations vs. population predictions (PRED) and the right shows that observations vs. population predictions logarithm, the black solid line is the line of unity y=x


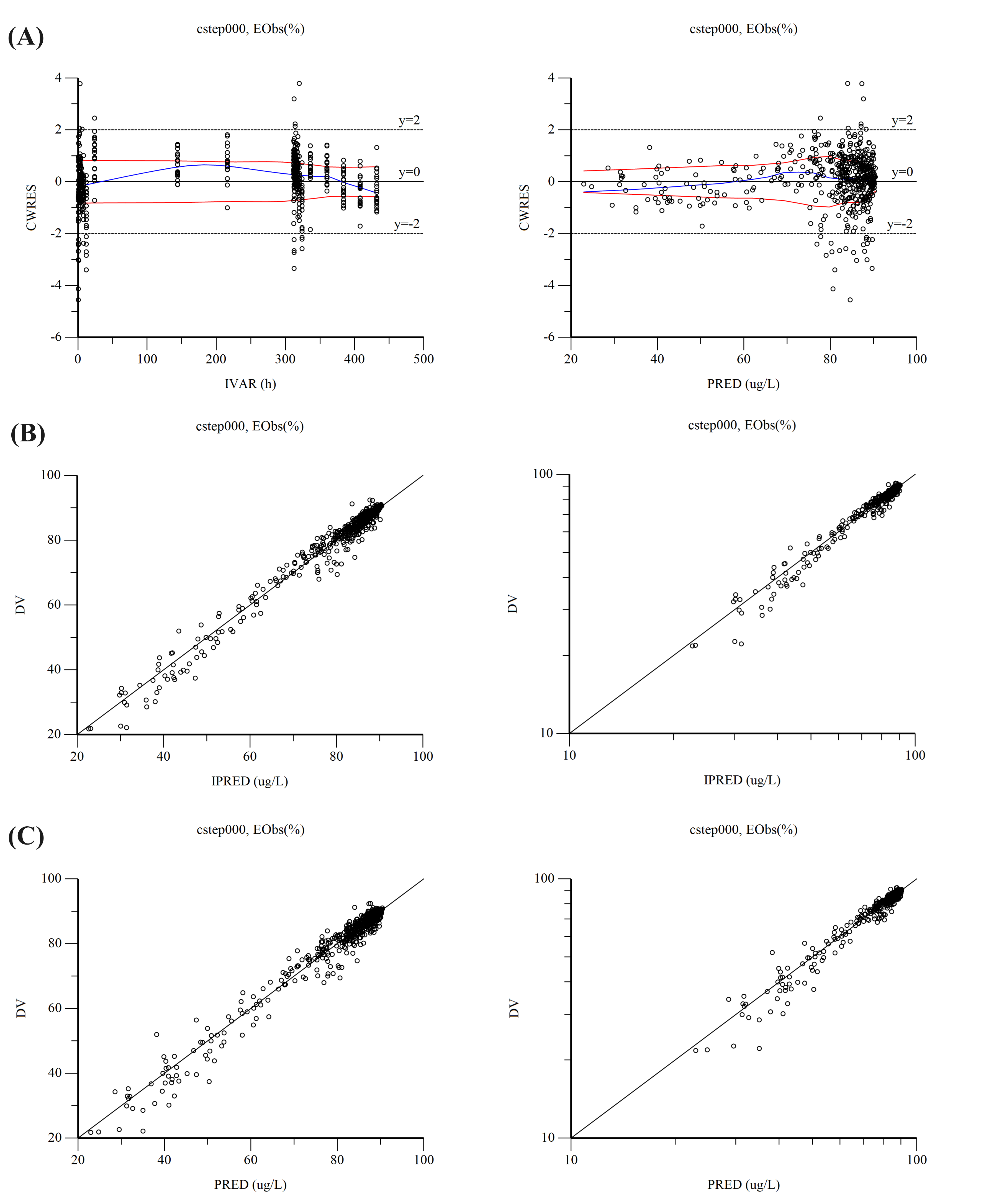


## Supplementary Tables

**Supplementary Table 1.** Parameter estimates and bootstrap results of the final population pharmacokinetic model.

| Parameter | Final model | | | | Bootstrap | |
| --- | --- | --- | --- | --- | --- | --- |
|  | Estimate | RSE (%) | 95%CI | Shrinkage (%) | Median | 95%CI |
| Population pharmacokinetic parameters | | | | |  | |
| tvK_a_ (1/h) | 0.06521 | 1.623 | 0.06313~0.06729 | NA | 0.06496 | 0.05856~0.07063 |
| tvV (L) | 8.668 | 2.234 | 8.287~9.048 | NA | 8.668 | 5.815~13.23 |
| tvV_2_(L) | 558.0 | 2.023 | 535.8~580.2 | NA | 561.6 | 480.2~651.6 |
| tvK_m_ (µg/L) | 171.5 | 6.694 | 149.0~194.1 | NA | 169.6 | 116.1~247.2 |
| tvV_max_ (µg/h) | 9373 | 5.044 | 8444~10302 | NA | 9294 | 7011~12491 |
| tvCl_2_ (L/h) | 9.671 | 2.050 | 9.281~10.06 | NA | 9.671 | 7.523~11.84 |
| dV_2_dTBIL | 0.3723 | 3.066 | 0.3599~0.3947 | NA | 0.3618 | 0.1417~0.5453 |
| Population pharmacokinetic/pharmacodynamic parameters | | | | |  | |
| tvEC_50_ | 5.120 | 3.850 | 4.733~5.507 | NA | 5.120 | 4.716~5.482 |
| tvG_am_ | 1.008 | 4.296 | 0.9234~1.094 | NA | 1.008 | 0.9042~1.080 |
| tvE_max_ | 91.78 | 0.6484 | 90.61~92.95 | NA | 91.78 | 90.83~93.16 |
| Inter-individual variability | | | | |  | |
| ω^2^V | 0.8112 | 8.340 | 0.6786~0.9438 | 18.24 | 0.7744 | -0.0281~1.647 |
| ω^2^V_max_ | 0.0260 | 4.482 | 0.02368~0.02824 | 8.642 | 0.02594 | -0.0012~0.0545 |
| ω^2^K_a_ | 0.01462 | 2.673 | 0.01385~0.01538 | 11.31 | 0.01355 | 0.0061~0.0210 |
| ω^2^V_2_ | 0.02874 | 1.115 | 0.02811~0.02937 | 12.68 | 0.02819 | 0.0077~0.0503 |
| ω^2^K_m_ | 0.01278 | 1.622 | 0.01237~0.01319 | 43.52 | 0.01081 | -0.0156~0.0410 |
| ω^2^EC_50_ | 0.01259 | 57.07 | -0.001493~0.02668 | 18.13 | 0.01259 | 0.0013~0.0253 |
| ω^2^E_max_ | 7.262E-05 | 58.50 | -1.065E-05~0.0002 | 17.58 | 7.26E-05 | 5.071E-06~0.0001 |
| Multiplicative residual variability PK(σ) | | | | |  | |
| Stev0 | 0.2241 | 5.429 | 0.2002~0.2480 | NA | 0.2230 | 0.1939~0.2521 |
| MixRatio residual variability PD(σ) | | | | |  | |
| Stev0 | 11.62 | 22.63 | 6.451~16.78 | NA | 11.62 | 7.897~18.21 |

RSE, relative standard error; CI, confidence interval; tvK_a_, typical value of absorption rate constant (K_a_); tvV, typical value of volume of central compartment distribution (V); tvV_2_, typical value of volume of peripheral compartment distribution (V_2_); tvK_m_, typical value of michaelis-menten constant (K_m_); tvV_max_, typical value of maximum rate of reation (V_max_); tvCl2, typical value of intercompartment clearance between the central and peripheral compartments (Cl_2_); dV2dTBIL, fixed parameter coefficient of total bilirubin (TBIL) to V_2_; tvEC_50_, typical value of concentration for 50% of maximal effect (EC_50_); tvGam, typical value of Hill coefficient (G_am_); tvE_max_, typical value of maximum pharmacodynamic effects (E_max_); ωV, variance of inter-individual variability for V; ωV_max_, variance of inter-individual variability for V_max_; ωK_a_, variance of inter-individual variability for K_a_; ωV_2_, variance of inter-individual variability for V_2_; ωK_m_, variance of inter-individual variability for Km; ωEC_50_, variance of inter-individual variability for EC_50_; ωE_max_, variance of inter-individual variability for E_max_; stdev0, standard deviation
